# Motorist’s Disorientation Syndrome—A Narrative Review

**DOI:** 10.3390/jfmk11020229

**Published:** 2026-06-03

**Authors:** Georges Dumas, Pierre Denise, Art Mallinson, Enrico Armato, Hannes Petersen, Philippe Perrin

**Affiliations:** 1 Research Unit DevAH—Development, Adaptation and Handicap, Faculty of Medicine, University of Lorraine, 54500 Vandoeuvre-lès-Nancy, France; georges.dumas10@outlook.fr (G.D.); armato.otovest@gmail.com (E.A.); 2 Department of Oto-Rhino-Laryngology Head and Neck Surgery, University Hospital, 38043 Grenoble, France; 3 Inserm, COMETE U1075, CYCERON, Université de Caen Normandie, CHU de Caen, 14000 Caen, France; pierre.denise@unicaen.fr; 4 Division of Otolaryngology, Department of Surgery, Faculty of Medicine, University of British Columbia, Vancouver, BC V6T 1Z4, Canada; artmallison@gmail.com; 5 Department of Neurosciences, University of Padova, 35100 Padova, Italy; 6 Department of Anatomy, Faculty of Medicine, School of Health Sciences, University of Iceland, 102 Reykjavik, Iceland; hpet@hi.is; 7 Department of Surgery, Akureyri Hospital, 600 Akureyri, Iceland; 8 Laboratory for the Analysis of Posture, Equilibrium and Motor Function (LAPEM), University Hospital of Nancy, 54500 Vandoeuvre-lès-Nancy, France

**Keywords:** motorist disorientation syndrome, visual vertigo, visio-visual conflict, visio-vestibular conflict, vestibular tests, optokinetic nystagmus, velocity storage integrator, cognitive behavioral therapies

## Abstract

Motorist’s disorientation syndrome (MDS) is seen in 1 to 5% of patients in a tertiary neurotology clinic and remains an underdiagnosed pathology. It was first described in 1985 by Page & Gresty, using the term “visual vertigo”. Patients described sensations of veering or turning over while driving an automobile when visual input was restricted. This was exacerbated at high speeds, on winding roads, going down hills, or when overtaken by a vehicle. All patients in this initial study had peripheral or central neurotological abnormalities and showed exaggerated responses during optokinetic stimulation. Some sufferers considered giving up driving. The first aims of this narrative review were to delineate the symptoms of MDS as detailed in the literature, to outline precipitating situations and to discuss associated pathologies such as anxiety. The second aim was to differentiate MDS from similar syndromes, such as persistent postural-perceptual dizziness (PPPD) and motion sickness (MS). In addition, we looked at the role of vestibular assessments and discussed the involvement of the otolith organs and semicircular canals. In this review, eight publications were analyzed. MDS is related to a visual-vestibular or a visio-visual conflict and occurs in drivers (both males and females). It is associated with anxiety in 17–39% of cases. Mild vestibular-test abnormalities or exaggerated response to opto-kinetic stimulations are seen in 60–100% of cases. Between 50 and 62% of patients have a migraine history. Convergence and strabismic problems are also often seen. Symptoms usually settle after 6 ± 4 years but can persist for longer in females. MDS is multifactorial, and similar to certain forms of PPPD but different than MS. Its pathophysiology is still in question, and we support the role of the velocity storage integrator as a recent hypothesis. Treatment includes vestibular rehabilitation, virtual reality, cognitive behavioral therapies and orthoptic sessions, and the results are promising. The authors also strongly feel that future research on clarifying MDS pathology should study a wider scope of vestibular assessments to evaluate semicircular canal/otolithic function, as well as the vestibulo-ocular reflex, analyze optokinetic nystagmus time constant, and perform a systematic orthoptic examination.

## 1. Introduction

The “motorist vestibular disorientation syndrome” (MVDS) was first described by Page and Gresty in 1985 [1] and has more recently been labeled “motorist disorientation syndrome” (MDS) [2]. It is a rare entity which is underdiagnosed and often not recognized. It occurs in patients with a strong visual dependency and is part of the entity sometimes referred to as visual vertigo. This entity has still not been totally clarified, but it is part of a functional vestibular disorder whose principal archetype is represented by persistent postural-perceptual dizziness (PPPD) [3]. PPPD is a chronic entity which is not structural and not psychiatric, typically favored by anxiety. It is characterized by unsteadiness and discomfort of variable intensity during the day related to standing up and walking, and it is often precipitated by complex visual stimulations. It constitutes the archetype of functional vestibular pathologies and includes different groups of previously isolated pathologies, such as phobic postural vertigo, visual vertigo, and chronic subjective unsteadiness. It is classically attributed to a dysfunction of the connectivity among the different cortical vestibular areas [3]. The two disorders have some common aspects and may present some overlapping symptoms and context (visual vertigo, visual dependence), but the individuality of MDS has been recently outlined in the literature by Ainsworth et al. [4] and Pawar et al. [5]. These authors emphasized that the dizziness and disorientation primarily occur in drivers, and is often associated with otolith anomalies or inappropriate use of sensory input. Motion sickness (MS) as defined by some authors [6,7,8,9] has been differentiated from MDS [5,6,7,8,9,10]. MS symptoms, which develop mainly due to a visual-vestibular conflict, are more rarely provoked by visual stimulation only. MS symptoms classically occur only in passengers due to a difference between actual and expected motion either in a car, on a plane, on a boat, in space travel, or when immersed in a virtual-reality situation. Symptoms commonly include nausea, vomiting, cold sweats, headache, dizziness, tiredness, loss of appetite, and increased salivation. This is a typical symptom set seen when children are reading in a moving car on winding roads.

With regard to differentiating MDS from MS, MDS affects drivers but not passengers. This introduces the concept of prediction/anticipation. A cognitive process involved in the interpretation of a dynamic environment may lead to an anticipation of upcoming accelerations (optimizing integration of vestibular and neck proprioceptive signals), which may limit the occurrence and influence of sensory conflicts. One goal of this work was to determine whether this entity can be singled out and isolated.

Visual dependence associated with “vestibular omission” was discussed by Freyss et al. in 1994 [11], who outlined that some elderly individuals prefer to rely on vision. This concept was also expanded by Bronstein et al. [2,10] and Feller et al. [12] and corresponds to patients who become symptomatic and dizzy in supermarkets and moving spaces.

MDS occurs in individuals who rely mainly on vision [2,10]. These individuals often also experience anxiety [4,5]. The incidence of the pathology is probably underestimated, since patients who are worried they may have to give up driving will not always discuss their symptoms with a clinician [10]. As some sufferers are particularly disabled in their daily life by their symptoms, they often search for healthcare solutions. There are some discrepancies in the literature that must be clarified, as some patients also experience symptoms when not in their car [4,5].

The question concerning the usefulness of documenting vestibular dysfunction using assessments, such as caloric tests, otolithic assessments, subjective visual-vertical testing or optokinetic nystagmus (OKN) assessment, remains open. The site of vestibular dysfunction in MDS patients is also unclear. Precipitating conditions such as a curved road or the summit of a hill suggest a possible otolithic contribution [1]. Alterations of OKN or optokinetic after nystagmus (OKAN) [1,4] and an unpleasantly increased circular vection during OKN stimulation [1] infer the involvement of the velocity storage integrator.

We have outlined the effect of anxiety and visual convergence impairment to clarify the pathophysiological implications of MDS with regard to visual-vestibular and also visio-visual contributions, as well as the contribution of the velocity storage system; this will be discussed later in this paper.

We also discuss possible treatment regimens for this disorder and particularly their current possibilities and insight into rehabilitation. Many heterogenous factors likely contribute to the development of this functional entity. This review is focused on the following three main goals: to extract from literature data the main characteristic symptoms, to differentiate this entity from other functional pathologies, and to provide significant results from vestibular explorations will aid in developing new insights for further pathophysiological conceptual hypotheses.

## 2. Materials and Methods

This narrative review collected the main clinical characteristics and epidemiological data related to MDS and was conducted with a transparent approach to the literature, analyzing observational studies and case series in order to subsequently discuss various pathophysiological hypotheses and treatments [13]. It did not follow a full systematic review protocol, as per the recommendations of the Preferred Reporting Items for Systematic Reviews and Meta-Analysis (PRISMA) checklist and reporting standards [14,15].

### 2.1. Search Strategy

In order to identify relevant studies on MDS, a keyword search was conducted across these various electronic knowledge bases, including PubMed, Scopus, Google Scholar, Embase, Web of Science, and Science Direct. Articles indexed were searched up to June 2025, and selection was performed following a similar PRISMA method (PROSPERO registry number: 1358002). A comprehensive literature search was conducted to identify relevant publications on MDS.

Search criteria included the following Boolean operators: [Motorist disorientation syndrome] OR [Motorist vestibular disorientation syndrome] OR [Highway syndrome]. [Visual vertigo] AND/OR [Visuo-vestibular induced vertigo], [Vestibular pathologies in drivers] AND/OR [Functional vestibular pathologies in drivers] [all fields].

Two independent investigators (GD and PP) screened the titles and abstracts retrieved from the databases. Duplicate records were removed prior to screening.

Disagreements regarding study eligibility were resolved through discussion between the two reviewers until consensus was reached. Only studies meeting the predefined inclusion criteria and agreed upon by both investigators were retained for further analysis.

### 2.2. Study Selection Criteria

Studies were included if they reported patients meeting the definition of MDS as originally described by Page & Gresty [1] and further characterized by Bronstein et al. [2,10], with clinical features clarified by Ainsworth et al. [4] and Pawar et al. [5].

Eligible publications included observational studies involving patients with MDS, case series describing the syndrome, and hypothesis papers or conceptual descriptions addressing the clinical entity.

Publications were excluded if the number of patients included was fewer than four, the clinical description did not correspond to the established diagnostic concept of MDS, insufficient clinical data were available to characterize the syndrome.

### 2.3. Data Extraction and Methodological Considerations

A total of 1947 publications were initially identified through the database search. After screening and application of the inclusion criteria, 1939 records were excluded.

Ultimately, eight publications met the inclusion criteria and were included in the final qualitative synthesis. These studies consisted primarily of case series describing patients with clinical features consistent with the original concept of MDS [1,2,4,5].

Given the limited number of available studies and the variability in study design, patient recruitment, and diagnostic criteria, a substantial degree of cohort heterogeneity was anticipated. Consequently, a quantitative meta-analysis was not performed, and the results were synthesized descriptively.

Potential sources of bias included small sample sizes, retrospective study designs, variability in diagnostic definitions, and publication bias related to rare clinical syndromes.

**Figure 1:**
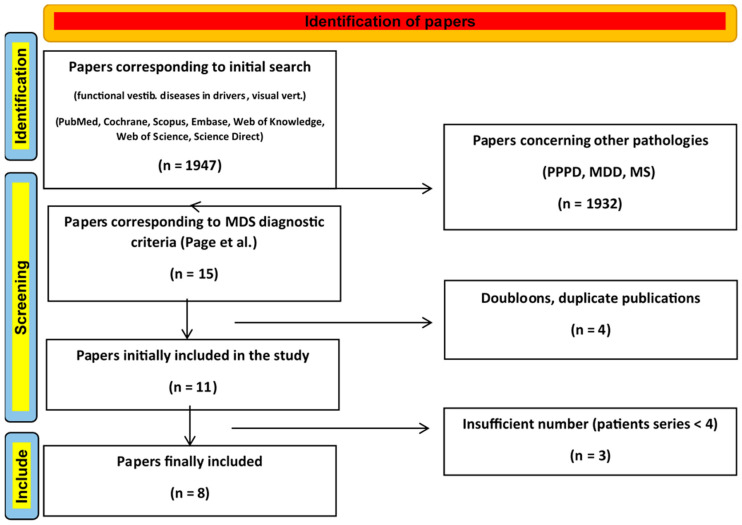
Flow chart illustrating how research into the identified articles was performed (adapted from the PRISMA method (Sukhera, 2022 [13]; Pgae et al. 2021 [14]; Veroniki et al. 2025 [15])).

The study selection process is summarized in the flow diagram presented in Figure 1.

## 3. Results

Results from the eight papers of the literature that remained following the flow chart process are summarized in Table 1.

A chronological presentation of these papers is detailed below and follows the historical individualization of this recent entity which remains rare possibly due to its under-identification.

The initial report by Page and Gresty in 1985 described the following:

Six patients (four males and two females), all who had differing levels of vestibular deficits (peripheral or central) [1]. There was no significant gender prevalence shown in this small series.

Symptoms: patients described an illusion of veering or turning into a vehicle or turning over under the following three conditions:

▯ Driving on highways at high speed (>45 mph) (usually in a monotonous, uniform landscape), which would induce a false sensation of veering (similar to a sensory-deprivation situation);
▯ Being overtaken by a truck, which would cause a sensation of veering toward the overtaking vehicle (induced by differences in visual flow);
▯ Being at the summit of a hill or going downhill and braking on a winding road, which would bring on a sensation of turning over.

Vestibular assessments in this group of patients were incomplete. Patients did not undergo head impulse test (VHIT), vestibular evoked myogenic potentials (VEMPs), or skull vibration induced nystagmus test (SVINT) (these tests, which are routinely carried out today [15], were not available at that time). However the vestibular and oculography assessments that were carried out showed abnormal results in all six patients.

OKN assessment was carried out in four patients. Caloric tests were carried out in two patients and rotatory tests in three other patients. Caloric and/or rotatory tests showed abnormalities in 4/5 patients. Caloric tests showed unilateral hypofunction and both caloric and rotatory assessments showed a directional preponderance. The OKN or OKAN was modified in 4/4 cases: the OKN was appropriately inhibited by fixation in 2 cases; 2 other cases showed a directional preponderance. However, in all patients, OKN was accompanied by an excessive circular vection; the OKAN usually showed hyperfunction.

Ocular pursuit was abnormal in 3/4 cases (usually hypometric with saccadic corrections or intrusions).

Despite not having tests that could detect otolithic pathology (SVV or VEMPS), Page and Gresty suggested that patients’ symptoms were “arising either from inappropriate signals from disordered vestibular canal and otolith organs or from a disordered central interpretation of vestibular information, which become manifest in the absence of adequate visual stabilization”. It can be seen how driving over the crest of a hill or changing direction when going downhill could produce “disordered otolith signals”, as suggested by these authors [1,16].

In a study presented in 2003 at the RTO HFM/Human Factors and Medicine Symposium, dedicated to spatial disorientation in military vehicle drivers, Gresty & Ohlmann reported that approximately 5% of patients seen in a neuro-otology clinic are diagnosed with MDS [17].

It was reported that there were about 10 MDS diagnoses per year, showing no clear gender prevalence.

The symptoms are similar to those previously described and are grouped into three categories (“the car feels as if it veers on wide, open roads; the car feels that it is turning into vehicles being overtaking; the car feels that it is about to turn over when descending or rounding a bend”) [17]. The triggering conditions are also similar.

These authors also emphasized that vestibular assessments in these patients are often normal or near normal, and they outlined the similarities between these patients’ complaints and those of airline pilots on long flights. They suggested the presence of a “vestibulo-vestibular conflict” (i.e., a possible canal-otolith conflict), brought on by activities such as driving down hills or braking on corners. They outlined that drivers rely on stable landmarks (i.e., static cues), i.e., what is reliable in their environment. They suggested this is accomplished by identifying invariant patterns in the consequences of actions. The most reliable sensory input that drivers utilize is vision. However, in a situation where there are few, if any, reliable visual cues (similar to pilots in clouds), visual estimates of distance orientation and heading may be impaired. The authors emphasized that a sensation of movement may, in fact, be real, but drivers may be experiencing a heightened perception of the actual movement. Partly because of this, the authors suggested therapy based on cognitive reappraisal of environmental stability and desensitization to motion.

Bronstein et al. (1995) [18] described 15 patients with visual vertigo (VV). MDS was not regarded at that time as being different from VV. Five patients had symptoms mainly when driving in a car. Thirteen patients had an associated peripheral disorder, seven had abnormal caloric or rotational tests, and two had central nervous system (CNS) disorders. Posturography tests were abnormal in five patients. Four had strabismic symptoms (diplopia, squint surgery, and oculomotor weakness). The authors suggested that this was a heterogeneous syndrome with peripheral or central pathology with high visual field dependence and often strabismic symptoms in many patients, which could result in inappropriate postural reactions in environments with conflicting visual stimuli.

In a later study, Bronstein et al. 2013 [10] reported a series of four MDS patients (three males and one female) which they differentiated from patients with MS. They proposed replacing the term “motorist vestibular disorientation syndrome” (MVDS) with the term “motorist disorientation syndrome” (MDS). The incidence was estimated to be about 1% of all vestibular patients seen in their clinic (five patients per year).

The patients reported sensations of veering off the road when driving, particularly on open roads. This was exacerbated by the sight of passing vehicles and also included a sense of instability and veering when exiting roundabouts or being on high bridges. Some patients experienced a sensation that their car might roll over when they were negotiating a curve or going down a hill. This was a concern, as a driver might make inappropriate adjustments while driving, and some patients actually gave up driving.

In 2020, Bronstein et al. [2] distinguished between visual-vertigo (VV), MS and MDS in the context of chronic dizziness and motion sensitivity.

In their population, they reported a series of six patients (five males, one female), which represented 1% of new patients they saw. They suggested that this pathology might possibly be underdiagnosed, since patients might be reluctant to seek medical opinion for fear of losing their driver’s license. They outlined that patients with normal vestibular function could still be affected by environmental visual challenges. In the presence of vestibular pathology, patients might rely on previous environmental experience and on visual stimuli. This visual dependence might result in symptoms, especially in the presence of anxiety. Patients related recent stressful episodes prior to the onset of symptomatology (two patients had had divorces and one had been on a plane that nearly crashed).

Symptoms and triggering situations: in this study, patients reported symptoms of veering and a sensation of a tilted horizon when rounding a bend (due to otolith ocular counter-rolling). The authors suggested that this could be generated by a differential in visual flow at high speed and noted that rapid optic flow from nearby had a higher angular velocity than visual input that was more distant. Patients also reported a sensation of tilting or rolling brought on by passing trucks. This was often interpreted as a slipping sensation and could induce a sensation of movement in the opposite direction (resulting in a potentially unsafe driving maneuver). In some cases, these inappropriate perceptions were so convincing that patients had changed cars and then realized it was not a problem with their vehicle. These motorists also complained of generalized disorientation and felt like “dizzy drivers”.

Proposed treatment: the authors proposed rehabilitation based on flying disorientation and MS, including therapy aimed at desensitization and “framework retraining”, along with appropriate cognitive behavior therapy.

An initial publication of Bronstein in 2004 [19] laid the groundwork for the three subsequent studies discussed [2,10,18] but will not be detailed here, as it did not detail any patient cases but was a discussion of the pathophysiology of VV. We will discuss this subject later in this review.

Guerraz et al. (2001) [20] carried out a prospective study that included the following:

twenty-one patients with VV (eleven females, ten males; mean age: 41). Patients with CNS pathology were excluded. Nine of them described vertigo when driving related to MDS.

Seventeen of the patients (81%) had an associated peripheral vestibular impairment. Some had a unilateral vestibular lesion (UVL).

Posturography was also carried out using visual stimulation (a moving landscape or rotating disc). Postural sway with eyes open (EO) was significantly modified in VV patients (including MDS patients) vs. normal patients and those with a UVL. The Romberg quotient (sway path with eyes closed (EC)/sway with EO) and visual kinetic quotient (sway path during disc rotation/sway path with EO) showed a significant increase in VV patients vs. normal subjects or UVL patients. The SVV, carried out using the rod and frame test and rotating disc, was also significantly impaired in VV and UVL patients compared to normal subjects, which could suggest a visual-vestibular mismatch and visual dependency. These authors suggested rehabilitation using OKN stimulation in order to reduce this visual preference.

Ainsworth et al. (2022) [4], in a retrospective study, described a large series of 18 patients [9 males; 9 females; age: 41.7 ± 10 (20–56)] with diagnosed MDS. They used the Dizziness Handicap Inventory (DHI) and Vertigo Symptom Scale (VSS), as well as a symptom questionnaire. Duration of symptoms was 6.39 ± 4.6 years and was longer in women. All included patients had vertigo when driving; 72% had vertigo only when driving. However, 62% of their patients also reported dizziness as a passenger, and 44% reported occasional vertigo when they were not in a car. Nine of the patients had a history of classical migraine.

The symptoms were similar to those reported by Page and Gresty [1]: 72% of their patients reported symptoms on highways at high speeds and 33% when going over hills. In 17 patients, symptoms were severe enough to cause a change in driving habits, and 6 of them gave up driving completely. Anxiety (VSSa score) was higher than normal and similar to that observed in vestibular migraine. These populations were compared to controls.

Vestibular tests (Table 1) (gaze, smooth pursuit, saccadic testing, bilateral caloric testing, impulsive rotation and OKN and OKAN analysis) were performed. However, there was no report of VHIT, VEMPs or SVINT assessments. Vestibular assessments carried out on 15 patients showed a 60% rate of abnormality. No modifications of smooth pursuit or saccade test were reported. Unilateral or bilateral caloric test abnormalities were observed in 5/15 cases and rotatory tests showing a directional preponderance in a similar proportion. OKN and OKAN also showed a directional preponderance in 5/15 patients.

In a series of 23 patients (mean age: 45.7 ± 8.7 years), Pawar et al. (2023) [5] developed a set of diagnostic criteria. They also suggested that pathology might be underreported in the literature due to a misrecognized or underrecognized entity. They limited their study to patients in a car and excluded open-air, two-wheeled vehicles. They used precise questionnaires about symptoms.

**Table 1:** Patients’ general data (population included). Series and characteristics. Accompanying pathologies. Evolution of symptoms. Age Me = age mean value; Age SD = age standard deviation; Age [] = age limits; Gender: M = male, F = female; Nb = number of patients; ND = no data; PVC = poor visual cues; Associated MS = motion sickness (MS)-associated pathology; Migr. = migraine; VV = visual vertigo; MDS = motorist disorientation syndrome; DHI = dizziness handicap inventory; DHI-T = total score; DHI-E = emotional; DHI-F = functional; VSS = vertigo symptoms scale; VSS-AA = autonomic, anxiety; VSS-VB = vertigo/balance disorders; Vestibular Explorations: CaT = Caloric test; ROT = rotatory test; OKN = optokinetic test; OKAN = optokinetic after nystagmus; SVV = subjective visual vertical; VNG = videonystagmography; CNS: central nervous system pathologies; OCM: oculomotor muscle implications. The importance of anxiety, visual susceptibility & functional impairment is evaluated by “+”: important and “++”: very important.

Authors	Series Nb Patients Study Design	Age Me SD []	Gender	Conditions - Vection - Ocular anomalies (Squint)	Anxiety	Migr.	Associated MS	Visual susceptibility (e.g., in crowd)	Functional impairment (Stop driving) DHI (Mean) VSS (Mean)	Vestibular explorations performed	Evolution (in years)
**Page & Gresty** (1985) [1]	MDS = 6 Retrospective study	51 [43–52]	2 F 4 M	(1) Veering highways high speed PVC, (2) turning (overtaking), (3) turnover (descending & rounding a bend)		ND		+	Modifications driving habits = 6 1 abandoned driving	OKN = 4 CaT-ROT = 5 Oculography (pursuit) 4	Duration: 2 yrs: 1 case 4 yrs: 4 cases 15 yrs: 1 case
**Gresty & Ohlmann** (2003) [17]	10 cases/200 consultations/year Retrospective study	ND	ND	Restricted sight in car or military vehicles	+ Cause/consequence	ND	Possible Unremarkable	+	Modification driving habits Untoward consequences		ND
**Bronstein** (1995) [18] Visual vertigo, some when driving	VV &/or MDS = 15 MDS = 5 (5 patients in cars) Retrospective study	39 [21–57]	ND	Vertigo Balance dis. in visual moving fields CNS = 2 (CT scan) 5 OCM dysfunction	ND	ND	ND	++	ND	Pursuit OKN VOR CaT Posturography	0.5 to 20
**Bronstein** (2013) [10]	MDS = 4 Incidence 1% vestibular consultation Retrospective study	ND	3 M 1 F		+	ND	ND	+	+	Posturography CaT	ND
**Bronstein et al.** (2020) [2] Visual vert. & MDS & MS	MDS = 7 Retrospective study	ND	5 M 1 F	- Veering highways - Turning overtaking - Turnover (descending & rounding a bend	+ (4/6) or phobia	ND	ND	+	1 car accident No specific guidelines on fitness MDS and driving		ND
**Guerraz et al.** (2001) [20] 21 Visual vertigo 9 MDS	VV = 21 MDS = 9 Prospective study	41 [25–60]	10 M 11 F	Squint: 14%	++ 43%	+ 24%	+	+		SVV (disc mvt) vs. controls Posturography (rotating disc) vs. UVL and controls	
**Ainsworth et al.** (2023) [4]	MDS = 18 Retrospective/Prospective study	41.7 10 [20–56]	9 M 9 F	Disorientation High speed Highways Id Gresty et al. But also out of car (50%) and when passenger (60%)		+ 50%	+		DHI-T = 34.6 8 = mild 6 = moderate 2 = severe DHI-E = 11.7 DHI-F = 12.8 VSS-T = 40 VSS anx. = 25 33% abandon driving	ENG Rotatory test Oculography (pursuit) OKN (no VHIT)	6 ± 4.6 years [6 months to 18 years]
**Pawar et al.** (2023) [5]	MDS = 24 Retrospective/Prospective study	45.7 8.7	ND	Id + looking at other vehicles or signs 42%	34%	62%	50%	+	+		[8 days to 10 years]

Symptoms and triggering conditions: Disorientation was reported in 79.2% of patients, but only when driving. Specific precipitating conditions included high speeds (>80 km/h) (67%), multilane roads (58%), bends and turns (50%) and looking at other vehicles or road signals when driving (42%). Patients were bothered by stimuli, such as overtaking vehicles, looking down from bridges, or driving in closed tunnels. Similar symptoms such as dizziness in open or overcrowded places (supermarkets and malls) were reported by 23% of patients (i.e., suggesting visual dependence).

Autonomic symptoms were also described, as follows: palpitations (41%), sweating (25%), cold extremities (4%).

The authors mentioned a history of migraine in 62.5% of their MDS patients, an associated MS in 50%, anxiety in 34%, and depression in 16%. The intensity of symptoms and severity of dizziness reported by patients on an analog scale of 1–10 was 6.1 ± 2.

The vestibular assessments carried out in 17 of the patients showed no anomalies for horizontal saccades and rare abnormalities for vertical saccades (hypometric in 12%) and pursuit. The authors discussed a migraine, which was often associated, and they proposed an antimigraine treatment in a number of these patients.

## 4. Discussion

Following the literature analysis we carried out (summarized in Table 2 and Table 3), the main points are synthesized regarding the MDS patient population, symptoms, triggering conditions, measured abnormalities and differential diagnosis in order to define this underrecognized entity. Table 4 is a conceptual summary of pathophysiological hypotheses. Meanwhile the literature data converge on triggering conditions such as visual vertigo and high-speed conflicting visual flow, and many papers outline difficulties in differentiating MDS from MS or PPPD. Objective vestibular assessment reports are either incomplete or do not specify canal, otolith or OKN perturbations; this aspect of our literature search will be discussed.

This recently described functional vestibular pathology has not yet been completely defined by the Barany Society in the International Classification of Vestibular Disorders (ICVD) [21] but is part of the three other functional diseases currently included in the ICVD (PPPD, Mal de Débarquement syndrome, MS). Specific rehabilitation programs need to be developed for this newly identified symptom set, and its medical treatment is outlined in Table 5.

### 4.1. Triggering Circumstances and Symptoms (Table 1 and Table 2)

As outlined by Bronstein, a type of motorist disorientation occurs in some individuals who develop a false perception of vehicle orientation [2,10], and this can result in the development of symptoms. In their seminal study, Page & Gresty [1] described a VV associated with dizziness or disorientation, experienced as illusions of turning over or veering while driving with reduced visual input. This was exacerbated at high velocity (around 45 mph) on highways, going around corners, going over the brow of hills, going down a hill, or when being overtaken by large vehicles. These symptoms are suggestive of visio-visual conflict, possibly precipitated by otolithic disfunction (Gresty & Ohlman) [17].

Pawar et al. [5] described other provoking stimuli, such as looking at traffic signals, driving over 80 km/h, or going through a tunnel or over high bridges. Chin [22] emphasized the role of eye focusing difference and convergence when moving from a dashboard display to the surrounding landscape.

The disorientation can often result in consequences such as drifting outside of one’s lane when driving. The potential of an accident did lead to some patients giving up driving [1,17]. This illusion of disorientation is significantly more frequent at high speeds on highways but is often reduced or extinguished at low speeds in the city. Gresty et al. suggested this may be due to a paucity of nearby visual cues in a large open landscape, similar to a pilot’s disorientation caused by a lack of accurate visual input at high altitudes [18]. As outlined in the literature, these patients present a visual preference or dependence (sensitivity to heights; movement in crowds, malls, or supermarkets; and stimuli such as passing cars or disco lights) [1,23].

One main precipitating circumstance is overtaking (or being overtaken by) another vehicle on the road. This visio-visual conflict was described by Gresty, Ainsworth and Pawar et al. [1,4,5,17]. It could be also a factor in rally-car drivers when seeing two different visual fields at high speed (for example a line of proximal trees on one side and distant landscape features on the other side) (PP personal report).

Accordingly, different situations can evolve. As described by Gresty and Ohlmann (2002) [17], when a car driver is overtaken by a large vehicle (e.g., a truck), the driver develops a sensation of going backwards with respect to the overtaking vehicle. If there is an open landscape on the other side, the sensation is of going forwards. The resulting illusion of vection can make a driver feel as if they are moving toward the overtaking vehicle. The visio-visual conflict can provide an explanation for this. When overtaking a large vehicle on a highway, the visual input on the driver’s side is slower than the visual flow on the opposite side (an open landscape), thus producing a possible illusion of veering toward the large vehicle. As a result, the driver may inappropriately turn his steering wheel contralaterally to correct the apparent “veering”.

On the basis of the description of this phenomenon laid out by Gresty and Ohlmann [17], we propose an explanation of some car accidents in rally-car drivers. At high speed, a sudden change in the landscape on one side could be problematic. For example, while driving in a forest and looking at trees on both sides, a suddenly open landscape on one side (e.g., a lake) would contrast with the faster passing contralateral forest. This could provoke a sudden sensation of sway toward the open landscape, with an inappropriate response of a driver turning his steering wheel contralaterally. This may explain some rally driver accidents, especially in a tired driver who might have a decreased level of alertness and awareness of their surroundings. A similar disorientation in professional drivers is also discussed by Pawar et al. [5]. Anxiety was also reported in 17 to 39% of cases [4,5].

Panic attacks may be consequences or may be associated with MDS, and the symptoms may, under certain conditions, be difficult to differentiate in these pathologies, as suggested by Brookler [24].

### 4.2. Population (Table 1 and Table 2)

In the two available open-series papers that reported gender and mean age (*n* = 24), [1,4], the mean age was 43.9 years old (SD = 10.1), and the sex ratio was 13 males/11 females.

Ainsworth et al. showed an equal number of males and females in their 18 subjects [4]. The small predominance of males in other earlier papers may be that there were more male drivers.

In another independent closed series from Pawar et al. [5], the mean age was 45.7; SD = 8.7.

As discussed by Bronstein et al. [25] and Brookler et al. [26,27], this pathology is underrated.

**Table 2:** Symptoms & precipitating conditions. The importance of the different precipitating conditions, vection illusions & the contribution of the different types of conflict following the different authors appreciations is mentioned by “+”: important and “++”: very important.

	Conditions to induce symptoms—Precipitating conditions							Vection illusion		Type of conflict	
										Visio-visual	Visio-vestibular
Authors	Wide space (paucity of visual cues)	- High velocity. - Highways, two-lane roads, motorways. - Overtaking	Hill brows Descending & turning	Tunnels	Bridges	Others - Signs along road - Trees scrolling	Feeling of veering (wide, open roads) Paucity of nearby visual cues	Feeling turning into vehicles when overtaking at high speed	Feeling turning over when descending and rounding a bend	Conflicting visual flow (overtaking)	Visio-vestibular conflict? (associated peripheral pathologies)
**Page & Gresty** (1985) [1] Nb = 6 **Gresty & Ohlman** (2002) [17]	++	++ (45 mph)	++				++	++	++	++ (overtaking)	+
**Bronstein et al.** (1995, 2013, 2020 [2,10,18])	+	+			+	Exiting roundabouts	++	++	+	++	+
**Ainsworth et al.** (2023) [4] Nb = 18	+	+ 72%				Hills, bumps 33%	+ 72%	?	?	+	
**Pawar et al.** (2023) [5] Nb = 23	?	++ (80 km/h)		+	+	Reading signs along road Looking at other cars	+	+		+	+
**Guerraz et al.** (2001) [20] Nb = 9 MDS Nb = 21 VV	+									+	+ 17/21

### 4.3. Vestibular Assessments (Table 3)

Semicircular canal function abnormalities (usually mild modifications) have been reported by Page and Gresty [1] and by Ainsworth et al. [4], with the most common one being unilateral hypofunction or directional preponderance in caloric or rotary tests. VHIT results (measuring vertical canals) and VHF assessments using SVINT were not reported. Although the complaints of the patients discussed by Paige and Gresty [1] suggested otolithic pathology, SVV tests and VEMPs were not available at that time to document this.

Later studies assessed patients with symptoms similar to those reported by Page and Gresty (i.e., suggesting otolith pathology) but these studies reported no VEMPs pathology, and only rare SVV abnormalities [20].

Interestingly Guerraz et al. [20], analyzing SVV in a context of dynamic visual perturbations, showed significant abnormalities in VV and probable MDS patients but not in a unilateral vestibular lesion (UVL) group. The suggestion was that VV patients have abnormally large perceptual and postural responses to disorienting visual environments. The symptom set was not necessarily related to anxiety or a past history of motion sickness but does manifest in vestibular patients if they have increased visual dependence and difficulty in resolving conflict between visual and vestibulo-proprioceptive inputs.

The role of the otoliths in MDS patients suggested by previous clinical work has not been specifically documented by SVV or VEMPs assessments.

On the basis of the two papers that discussed OKN assessment [1,4], we suggest a possible contribution of the velocity storage integrator (VSI) to this pathology.

Central exploration such as oculography modifications have been reported in patients with associated CNS pathology [1,5]. Pursuit modifications were described in three of four patients reported in these two papers. Horizontal pursuit alterations were seen in 24% and vertical pursuit alterations in 90% of patients. Pawar et al. [5] mentioned normal horizontal saccades but hypometric vertical saccades in 12% of patients.

**Table 3:** Vestibular explorations.

Authors (Ref.) Nb Patients	CaT	Rotatory test	SVV	VHIT VEMPs SVINT	OKN OKAN	Posturography	Pursuit Saccades	Other
**Page & Gresty** [1] Nb = 6	2/4 hypofunction 2/4 directional preponderance	2/3 directional preponderance	ND	ND	OKN = 4/4 directional preponderance OKAN = hyperfunction	ND	Ocular pursuit abnormal in 75% (hypometry with saccadic intrusions)	Excessive feeling of circular vection after OKN
**Bronstein et al.** [2,10] Nb = 15 VV (5 MDS) Nb = 6 MDS	mild modifications = 13 ND	mild modifications = 13 ND	ND ND	ND ND	ND ND	ND ND	ND ND	ND
**Guerraz et al.** [20] Nb = 21	modified in 17/21 (80%)	ND	Normal in basic visual conditions	ND	ND	Normal in basic conditions without external visual stimulation		SVV is modified in dynamic conditions (rotating. disc) and external visual stimulations. Posturography modified by disc rotation
**Ainsworth et al.** [4] Nb = 18 (15 performed all the tests)	3 unilateral canal paresis 2 bilateral hypofunction		ND	ND	2 OKN: directional preponderance 2 OKAN: directional preponderance	ND	ND	ND
**Pawar et al.** [5] Nb = 24	ND	ND	ND	ND	ND	ND	Hor. pursuit normal Vert. pursuit: 12% Hor. sacc.: 24% Vert sacc.: 90% (hypometry)	ND
**Total**	**26/45** **(57%)**	**2/3** **(70%)**	**Normal** (in basic condition)	**ND**	**8/19** **(42%)**	**Normal** (in basic condition)	**(12% to 70%)**	**Dyn. SVV: VV** **≠** **Ct: *p* < 0.01** **Dyn. post: VV** **≠** **Ct: *p* < 0.01**

CaT = bithermic caloric test; SVV = subjective visual vertical; VV = visual vertigo; VEMPs = vestibular evoked myogenic potentials; SVINT = skull vibration-induced nystagmus test; OKN = optokinetic nystagmus; OKAN = optokinetic after nystagmus. ND = no data.

### 4.4. Proposed Characteristics to Separate MDS from Other Functional Pathologies

#### 4.4.1. Motion Sickness

In patients having a visual dependence, MDS and MS may both present with similar symptoms of discomfort occurring in a car.

However, Ainsworth and Pawar [4,5] have described some cases of MDS who present symptoms both as passengers and drivers and describe symptoms suggesting an associated MS in 50% of MDS patients. This outlines the difficulties encountered in trying to separate these two entities.

The sensory conflict theory is perhaps the most widely accepted theory of MS [28].

As discussed, a difference between MS and MDS is that MDS occurs when driving and cannot be diagnosed if symptoms are mainly observed as passengers or rally-car navigators.

A susceptibility to anxiety traits using the MS susceptibility questionnaire (MSSQ) [29,30] has been described by Paillard et al. [9] in MS patients but not in patients with vestibular loss. Bronstein et al. have stressed that vestibular function is usually normal in MS patients [2].

A cognitive process involved in the interpretation of the dynamic environment may lead to anticipation of upcoming accelerations, and this process can optimize the integration of vestibular and proprioceptive signals. The development of MS in a rally-car navigator could be explained by the constant gaze change between a notebook and/or map on his lap and the road ahead (i.e., outside environment). This requires frequent adjustments of the VOR gain, and the associated head movements could generate Coriolis accelerations [31]. This is a reasonable theory for the development of MS, but may also sometimes be a factor in MDS. For example, a driver on an unfamiliar road (perhaps relying on GPS) will need to change his gaze direction and head inclination repeatedly (in both pitch and yaw planes) from his dashboard GPS display to the external environment [31]. Coriolis acceleration side effects result from a head rotation about one axis during sustained body rotation about another axis and can precipitate MS [32]. The so-called “Coriolis effect” can also be disorienting. A discussion of this is beyond the scope of this paper, but this effect may increase symptomatology in MDS patients.

#### 4.4.2. Vestibular Spatial Disorientation and PPPD

Although some studies state that vestibular tests must be abnormal for MDS to be diagnosed [1], others mention that this diagnosis can still be made in patients with normal vestibular function and, as a result, differentiating between MDS and PPPD is difficult [4,5]. Some MDS patients (as many as 44%) also have symptoms when not in their automobile [4]. Otherwise in MDS patients, symptoms of veering or turning over are important and often different from the usual simple unsteadiness observed in PPPD patients, which is more common in females [3].

Differentiating PPPD from MDS can be difficult when patients have complaints in their vehicles, but also in other situations. In their diagnostic and inclusion criteria, Pawar et al. reported 24 patients who had symptoms only when driving [5]. However, we suggest that a diagnosis of MDS should be made if driving is the situation most likely to cause symptoms. Anxiety is often described and associated with psychological distress in PPPD patients, and this may precipitate symptoms (criteria C & D of a PPPD diagnosis) [3]. In MDS, anxiety traits are less frequent, but oculomotor or eye convergence anomalies are seen more frequently [2]. Gresty et al. suggested that differentiating MDS from phobic postural vertigo or agoraphobia was difficult in some patients [16].

#### 4.4.3. Drug Side Effects

Adverse effects of medication, toxic drugs (e.g., cannabis) and opioid byproducts may induce intrinsic oculomotor perturbations. The resulting visuo-vestibular conflict could precipitate symptoms of MDS. History taking in a patient can help rule out any drug side effects.

#### 4.4.4. Bilateral Peripheral Vestibular Impairment

Patients with significant bilateral impairments often report oscillopsia in vehicles but also when walking. Vestibular tests (calorics and VHIT) are helpful in ruling out any effects of coexisting bilateral vestibular pathology.

### 4.5. Pathophysiological Hypothesis of MDS (Table 4)

Table 4 is intended to be a conceptual summary of pathophysiological hypotheses.

MDS is a multifactorial heterogeneous entity and is part of the visual vertigo syndrome. Bronstein (1995) [18] and Guerraz et al. [20] detailed the influence of conflicting visual fields on posturography findings in patients with visual dependence, regardless of any CNS impairment or peripheral vestibular abnormalities. Symptoms of strabismus could result in inappropriate postural reactions in environments with conflicting or disorienting visual stimuli, likely by reducing the ability to resolve the sensory conflict.

In a study on visual-vertigo published in 1996 [33], Sawle et al. reported that visual vertigo on roller coasters and other fairground rides was more frequent in people with visual dependence (discomfort in malls, supermarkets, or crowded spaces). These patients are also made symptomatic by standing on posturography platforms or by being immersed in a moving visual landscape. They showed a significant sway increase, which the authors suggested were related to visual/vestibular conflicts, joint position and other sensory inputs (e.g., diplopia, extraocular muscle limitation, or history of surgery to correct a squint). This was also described by Bronstein (1995) [18].

Chin, in 2018 [21], suggested an oculomotor origin for this VV and emphasized the important role of vision and visual convergence in generating MDS. This article suggested that when drivers are looking straight ahead at the road and then gaze shifting to the dashboard, an erroneous convergence may have cervical postural consequences that could result in misleading postural interpretations. It is outlined that when an individual is shifting gaze or engaging in near point work, fatigue of ocular motor muscles (primarily superior rectus) is inevitable.

The possible role of the velocity storage integrator (VSI) is suggested by the studies of Gresty et al. [1] and Ainsworth et al. [4], who used OKN stimulations (although these authors did not explicitly cite the VSI system). We contend that a dysfunction of the VSI should be considered in more detail, in face of the important modifications of OKN and the after effects provoked by OKN stimulations in some individuals (duration of OKAN or nystagmus directional preponderance). These authors mentioned also that an optokinetic stimulus (full, or large-field visual motion) induced an unpleasantly increased sense of circular vection during stimulation. Optokinetic stimulation does not directly drive the OKN but is temporally integrated by the VSI located in the brainstem [34,35]. This extends and shapes the eye velocity response, as well as the false sensation of self-motion (vection). This response is beyond the physical duration and dynamics of the visual or semicircular canal stimulus [34,35,36,37,38,39]. As vection is increased in patients with MDS, we can hypothesize that, in these patients, the VSI reacts excessively in a dysfunctional manner.

This dysfunctional response can be connected to certain pathophysiological hypotheses already proposed in vestibular agnosia. In this condition, symptoms are also observed when walking, or turning one’s head when getting out of a car. These patients have a markedly inaccurate subjective estimation of the degree of rotation during rotatory testing. The peripheral vestibular function assessments evaluating the VOR (caloric tests, VHIT, VNG rotary test) are often normal. Symptoms are usually persistent and observed in vehicles but also in other environmental situations [40].

This suggestion that the VSI inappropriately contributes to development of symptoms in other functional pathologies such as mal de Débarquement syndrome has recently been advanced in the literature (Maruta et al.) [41].

The hypothesis of VSI in MDS could be tested and further checked by measuring the VOR time constant (the marker of VSI function) which could be prolonged in MDS patients. It is Interesting to note that a positive correlation between susceptibility to MS and the VOR time constant has been observed [42,43], which again highlights the link between MDS and MS.

Anxiety has been described as favoring a “peripersonal space” dependency (the space immediately surrounding one’s body) by Holmes [44] and D’Angelo [45]. This could possibly create an early visual dependency in younger people, unrelated to what is classically described in the elderly, who develop visual dependency linked with aging [11].

**Table 4:** Pathophysiological hypothesis; VV: Visual vertigo; VSS AA: Vertigo subjective scale, subsection autonomic, anxiety; ND: no data; NI: not informed (but performed); NS: not significant; dir. prep = directional preponderance. The intensity of the contribution of each item evaluated by the different authors is: “+” = important, “++” = very important.

Authors	Vestibular peripheral anomalies (Canal/Otolith)	OKN anomalies	Central anomalies (pursuit, others)	Oculomotor convergence	Visual dependence (e.g., supermarket)	Anxiety	Visual vertigo	Visio-vestibular mismatch	Visio-visual conflict	Multifactorial pathology	Velocity Storage Integrator contribution
**Gresty & Page** [1,2] Nb = 6	++ (canal/otolith conflict)	++	+	ND	+		++	+	?	?	?
**Bronstein** [2,10] Nb = 10	+	+		++ (squint surgery)	++		++			++	?
**Chin** [19]	ND	ND	ND	++	+		+		+		?
**Guerraz et al.** [18] VV = 21 MDS = 9	++ (17/21)	ND			++ SVV modific. by rotating disc ++ Tilted frame Postur. sway (rotat. disc)	++	++				
**Ainsworth et al.** [4] Nb = 1 8	+ CaT: 5 hypofunction ± dir. prep.	+	NI			++ (VSS AA)	++	+	+		?
**Pawar et al.** [5] Nb = 24	ND	ND	+ (migraine)	ND		+	+	+	+		
***Synthetic suggestions from the authors of the review***				++	++	+	++	+	+	++	++

Accordingly, we propose to identify two groups of MDS, as already suggested by Pawar et al. [5], who described primary and secondary MDS, as follows:

1. A group without any vestibular involvement or measured test abnormalities. This suggests a pure “visio-visual” conflict (i.e., a differential lateral visual flow). For example, being overtaken by a vehicle at high speed on a highway will contrast with the visual information coming from the other side of the road. This visual discrepancy would create a sensation of displacement of one’s vehicle (this was detailed by Page & Gresty) [1]. However, it is difficult to differentiate this condition from PPPD, as these patients may exhibit symptoms of anxiety, and in addition their high visual dependence may also cause similar symptoms when they are not driving.

The question to be asked is, why do the symptoms occur? We suggest that this is caused by other conflicting conditions due to driving circumstances and functional visual abnormalities (eye convergence) related to the fact that a driver must constantly alternate gaze between his dashboard and the road ahead. Chin [21] suggested that this could result in an unadapted spatial perception of the environment and perhaps inadequate postural responses.

2. In the second group, there is still a visio-visual conflict but also an alteration in otolith or canal function which can then amplify the conflict. It may be difficult to differentiate this group from MS or PPPD, as patients with predisposing high visual dependency and/or anxiety may also complain of vestibular symptoms when not in their car. These patients have symptoms at the crest of hill or when driving downhill on a winding road [1]. There is either a visio-vestibular conflict due to objective alteration of canal or otolith function, or a distortion of peripheral vestibular information being fed in to the neural centers, possibly related to VSI. Sang et al. [46] have also suggested a possible consequence of derealization or depersonalization in patients with objective measures of vestibular dysfunction, which could potentially create disorientation in driving conditions. Difficulty driving in normal conditions may be the result of a real-world manifestation of impaired spatial cognition associated with vestibular loss. It is also suggested that this may be a marker of more severe vestibular dysfunction [47,48].

In summary, we propose restricting the diagnosis of the typical or definitive clinical presentation of MDS to drivers who only experience symptoms in four-wheeled vehicles with vision restricted (as outlined by Page & Gresty [1]) but not on two-wheeled vehicles [5]. In the context of anxiety and possible co-existing otolith perturbations and visual dependence, a driver with a visual convergence disorder might have difficulty when shifting gaze from the dashboard to the road. As mentioned previously, this could be exacerbated by Coriolis forces, for example, when shifting the head repeatedly from the landscape to the GPS on the dashboard (e.g., if a driver is traveling on an unfamiliar road).

A diagnosis of possible MDS could be made in patients with symptoms occurring mainly when driving, but also when not in their car (Ainsworth) [4]. These patients have MDS but also an associated VV or PPPD, which can make the differential diagnosis more difficult. Pawar et al. [5] reports an association with MS and occurrence of symptoms as passengers in 50% of their 24 MDS patients. These authors differentiate MDS from PPPD and VV by mentioning that the PPPD score (Niigata questionnaire) in their MDS patients ranged from 0 to 27 (median (Q1, Q3) score of 2.5 (0, 7.75)) and that none of their patients had a high enough visual score to qualify for a diagnosis of VV or PPPD.

We wondered why MDS does not usually occur when pilots are flying their airplane. The selection of pilots is extremely stringent and screens out people who have psychological issues, visual difficulties, or problems with convergence. In addition, a pilot’s environment is different from that of a motorist. Aircraft pilots are susceptible to experiencing common “physiologic” disorientation in the cabin due to Coriolis accelerations as described during the Second World War. Gresty & Ohlmann [17] described some similarities between MDS and pilots’ disorientation.

It is important to note that when vestibular function is altered, most of the studies we reviewed implicate the otolith system [1].

### 4.6. Therapeutic Strategies (Table 5)

#### 4.6.1. Medications

Although this was not a systematic double blinded study, antimigraine treatments have been proposed by Pawar et al. [4] with diverse results of beta blockers (Bisoprolol) showing partial favorable effect.

Anxiolytic (amitriptyline, venlafaxine, etc.) or antidepressant therapy (e.g., desvenlafaxine) have been described by Pawar et al. as showing variable results. In this study, about 30% of cases showed an apparent clear improvement, about 30% of cases showed a mild improvement, and the remainder showed no improvement [4]. These drugs may also have side effects which may impair the ability to drive.

#### 4.6.2. Rehabilitation

Optokinetic stimulations have been proposed by Guerraz et al. [20] to increase a patient’s use of vestibulo-proprioceptive cues. These arguments can be linked to results already presented by Vitte et al. [49], who used OKN stimulations to reduce visual dependency.

Ainsworth et al. [4] have suggested a combination of visual vertigo and gaze stabilization exercises.

Adapted software using virtual reality stimulations seems to be promising but must be used carefully. However it does correspond to the pathophysiological hypothetic premises proposed by Gresty and Ohlmann [1,17].

Some studies propose cognitive behavioral therapy, with an aim of desensitization to visual stimuli [1,2,17]. These desensitization strategies are also recommended by Ainsworth et al. [4] and Bronstein et al. [2]. It has been proposed by Gresty and Ohlman [17] to start with simple environment visual stimulations and then progress to increasingly unstable and ambiguous environments. In future, some novel approaches could include a tactile vibration system to overcome spatial disorientation as previously described in aircraft by Paillard et al. [9]. It must be kept in mind that vibration perception thresholds are correlated with age but not with pathology (VV or vestibular pathology) [20]. OKN rehabilitation is more effective in younger people [4] because that population is more likely to compensate effectively in the presence of co-morbid factors such as other vestibular disorders.

Orthoptic treatments have been recommended [22] and good results have been described in a short series [50] (although this was not a double-blind systematic study).

**Table 5:** MDS proposed treatments in literature.

Authors	Medical treatment	Vestibular rehabilitation
**Page & Gresty** (1985) [1] (6 cases)	No detailed data; no effect of Cinnarizine	
**Gresty & Ohlmann** (2003) [17]		Cognitive therapy and desensitization to motion
**Bronstein** (1995) [18] Visual vertigo in 15 cases, 5 when driving MDS	No data	Interest in orthoptic considerations Interest in learning to refer to more proprioceptive cues and ignore misleading visual cues
**Bronstein** (2013) [10] (4 cases)		Rehabilitation similar to flying disorientation and motion sickness
**Bronstein et al.** (2020) [2] Visual vert. & MDS & MS (7 cases)	Anxiety treatment	Cognitive therapy rehabilitation, desensitization to movement Postural relaxation
**Guerraz et al.** (2001) [20] 21 Visual vertigo 9 MDS		Optokinetic (OKN) rehabilitation
**Ainsworth et al.** (2023) [4] (18 cases)	Medical treatment of motion sickness; efficient in 1/2	Traditional vestibular rehabilitation: not effective (1/5) Visual-vertigo rehabilitation + gaze stabilization: effective in 50%
**Pawar et al.** (2023) [5] (24 cases)	Migraine medical treatment (amitriptyline, propranolol, topiramate, etc.) (good results in 40–80%) Pregabalin and gabapentin (70 to 90% improvement)	

### 4.7. Limitations

The scarcity of studies (eight studies with detailed case reports), the small sample sizes (4 to 23 patients) and the few research groups infer risks of bias. Articles specifically dedicated to MDS rehabilitation are rare and discuss the less specific topic of visual vertigo rehabilitation, which is outside the scope of this work.

## 5. Conclusions

Historically MDS is understood to be a multifactorial symptom set. Its incidence is somewhat rare but it is probably an underestimated entity. It is mainly linked to visual dependence and anxiousness and is seen in both males and females. Clinically, the symptoms that mainly refer to the initial seminal description offered by Page and Gresty [1] are essentially based on visio-visual conflict, which can induce sensations of veering or turning over at high speed or on curves or when being overtaken by large vehicles. In order to differentiate the symptom set from PPPD and MS, a diagnosis of MDS should be restricted only to drivers of four-wheeled vehicles who have experienced a change in visual input from their surroundings or peripersonal space while driving. It is frequently associated with mild objective vestibular deficits or impairments (most often otolith dysfunction). OKN alterations support a possible link to dysfunction of the velocity storage system or integrator. It may also be related to insufficient eye convergence and cervical linkage proprioceptive mechanisms. The role of Coriolis forces may also be a contributing factor in some circumstances. Two main forms can be delineated, as follows: MDS with no vestibular-test abnormalities (corresponding mainly to a visuo-visual conflict) and MDS with detectable vestibular pathology (suggesting a possible visual-vestibular mismatch). Its treatment modalities are not presently clear but should rely on optokinetic rehabilitation and prudent use of adapted virtual reality and cognitive behavioral therapies.

## Data Availability

No new data were created or analyzed in this study. Data sharing is not applicable to this article.

## Abbreviations

The following abbreviations are used in this manuscript:

CaT	Caloric test
MDVS	Motorist Disorientation Vestibular Syndrome
MDS	Motorist Disorientation Syndrome
PPPD	Persistent Postural-Perceptual Dizziness
MS	Motion Sickness
OKN	Optokinetic Nystagmus
VEMPs	Vestibular Evoked Myogenic Potential
VSI	Velocity Storage Integrator
VV	Visual Vertigo
